# Epidemiology of ESBL-Producing, Carbapenem-Resistant, and Carbapenemase-Producing Enterobacterales in Southern Africa

**DOI:** 10.3390/antibiotics15010069

**Published:** 2026-01-08

**Authors:** Pearl Ntshonga, Giacomo Maria Paganotti, Paolo Gaibani

**Affiliations:** 1 School of Allied Health Professions, Faculty of Health Sciences, University of Botswana, Gaborone Pvt Bag 0022, Botswana; 201702229@ub.ac.bw; 2 Botswana-University of Pennsylvania Partnership, Gaborone P.O. Box 45498, Botswana; paganottig@bup.org.bw; 3 Division of Infectious Diseases, Perelman School of Medicine, University of Pennsylvania, Philadelphia, PA 19104, USA; 4 Department of Diagnostics and Public Health, Section of Microbiology, University of Verona, Strada Le Grazie 8, 37134 Verona, Italy; 5 Microbiology and Virology Unit, Azienda Ospedaliera Universitaria Integrata Di Verona, 37134 Verona, Italy

**Keywords:** ESBL-producing, carbapenem-resistant, carbapenemase-producing, Southern Africa

## Abstract

**Background/Objectives**: Antimicrobial resistance (AMR) among Enterobacterales poses a major threat to public health in Southern Africa and has led to limited treatment options and increased mortality. Despite Africa bearing the brunt, there is limited data on the epidemiology and molecular epidemiology of the genetic determinants of β-lactam and/or carbapenem resistance. This narrative literature review summarizes the epidemiology and molecular characteristics of extended-spectrum β-lactamase-producing Enterobacterales (ESBL-PE), carbapenem-resistant Enterobacterales (CRE), and carbapenemase-producing Enterobacterales (CPE) in Southern Africa, while identifying data gaps and surveillance challenges. **Methods**: A comprehensive literature review was conducted using peer-reviewed articles from ten Southern African countries, including South Africa, Lesotho, Eswatini, Botswana, Namibia, Angola, Zambia, Zimbabwe, Mozambique, and Malawi, reporting the epidemiology and/or molecular characterization of ESBL-PE, CRE, and CPE. **Results**: ESBL-PE, CRE, and CPE pose an increasing healthcare threat in Southern Africa, with prevalence varying widely by source. *Klebsiella pneumoniae* and *E. coli* are the predominant ESBL-PE, CRE, and CPE species. The most frequent resistance genes are *bla_CTX-M_* among ESBLs and *bla_NDM_* and *bla_OXA_* among carbapenemases, reflecting global patterns. However, molecular characterization across the region remains limited, with countries such as Botswana, Lesotho, Eswatini, Zambia, and Zimbabwe lacking sufficient data on the prevalence and diversity of these resistance determinants. **Conclusions**: Despite the paucity of genomic and epidemiological data, Southern Africa faces an urgent AMR challenge. Strengthening laboratory infrastructure, genomic surveillance, and regional coordination is crucial to mitigate AMR and guide antibiotic stewardship policies.

## 1. Introduction

The development of multi-drug resistance, especially in Gram-negative bacteria, is a global health problem. In response, the World Health Organization adopted the Global Action Plan (GAP), which countries have adapted into their National Action Plans (NAPs) for combating antimicrobial resistance (AMR) [1]. The overall aim of this policy is to ensure continuity of effective and accessible treatment for bacterial infections by improving awareness of AMR, supporting AMR surveillance and research, optimizing antibiotic use, and reducing the incidence of bacterial infections [1]. However, implementation and funding for NAPs have been uneven by member countries, leading to uncertainties regarding achievement of the goals of the NAPs.

The Southern African region, which consists of the countries South Africa, Lesotho, Eswatini, Botswana, Namibia, Angola, Zambia, Zimbabwe, Malawi, and Mozambique, had the fourth highest rate of deaths attributable to and associated with bacterial antimicrobial resistance, with more than 70 deaths per 100,000 people [2]. The age-standardized mortality rate in this region was higher than 75 deaths per 100,000, representing one of the highest mortality rates globally [3]. Despite this region bearing one of the highest AMR-associated and AMR-attributable deaths [3], countries such as Angola and Mozambique are significantly challenged in implementing their NAPs, while South Africa, Zambia, and Zimbabwe are leading the WHO Africa region with functional formalized multisector coordination mechanisms, funding and reporting arrangements for their NAPs [4].

Infections caused by multi-drug-resistant bacteria were estimated to account for 4.95 million deaths worldwide, with the numbers disproportionally higher in low-middle income settings (LMIC). There were an estimated 1.05 million deaths associated with and 250,000 deaths attributable to AMR in the WHO African region in 2019 [2]. This places the WHO African region as having the highest fatal and non-fatal burden of AMR among all regions. The leading fatal infections were as follows: lower respiratory infections (119,000 deaths), bloodstream infections (56,000 deaths), intra-abdominal infections (26,000 deaths), and tuberculosis (18,000 deaths).

The leading bacterial pathogens included *Streptococcus pneumoniae* (39,000 deaths), *Acinetobacter baumannii* (48,000 deaths), *Klebsiella pneumoniae* (50,000 deaths), *Escherichia coli* (37,000 deaths), and *Staphylococcus aureus* (30,000 deaths) [2]. The pathogen–drug combinations in the WHO African region with the highest AMR associated mortality were third-generation cephalosporin (3GC)-resistant *K. pneumoniae* (19,000 deaths), trimethoprim-sulfamethoxazole (TMP/SMX)-resistant *S. pneumoniae* (16,500 deaths), and methicillin-resistant *S. aureus* (15,300 deaths) [2]. Notably, deaths attributed to resistance to β-lactam/β-lactamase inhibitor (BL/BLI) combinations and carbapenems were higher in *K. pneumoniae* (2140 and 4610 deaths, respectively) and *E. coli* (3680 and 3140 deaths, respectively).

In 2019, the number of AMR-attributable deaths was 15,300 in the southern sub-Saharan African region, corresponding to 19.4 deaths per 100,000 [5]. According to a 2024 retrospective analysis of AMR in Africa, which included four countries from Southern Africa (Eswatini, Malawi, Zambia and Zimbabwe), the prevalence of extended-spectrum β-Lactamase (ESBL) and carbapenem-resistant Enterobacterales (CRE) has been on a steep incline, with the highest rate of resistance to β-lactams recorded in Zambia in 2019 (72%), and the highest rate of resistance to carbapenem drugs recorded in Zimbabwe in 2018 (8%) [6]. Indeed, the spread of ESBL and CRE represents a critical global public health threat, leading to increased morbidity, mortality, length of hospital stays, and significant healthcare costs. For example, in a case–control study in Senegal (West Africa), bloodstream infection (BSI) caused by ESBL-positive Enterobacterales was associated with higher case fatality rate than ESBL-negative BSI, with the case fatality rates being 54.8% vs. 15.4%, respectively [7]. Moreover, the multistate modeling analysis indicated that ESBL production was associated with an excess length of stay of 4.3 days [7]. Similarly, CPE have been associated with long hospital stays in South Africa, with New Delhi Metallo-beta-lactamase (NDM) production leading to 44.0 days vs. 13.3 days and 4 times longer intensive care unit (ICU) stays [8]. The economic impact of AMR, inclusive of ESBL and CPE, is estimated to cost worldwide health systems from USD 300 billion to more than USD 1 trillion annually by 2050 [9], which makes AMR a particularly challenging problem for LMICs. The Centre for Global Development (US) model estimates the cost-per-admission for MDR non-TB bacteria in LMICs at approximately USD 1000, which is 2.2 times higher for resistant bacteria in comparison to susceptible bacteria [10], whereas the same disparity is only 1.4 times in high income settings. In the Southern African region, estimates of hospital admissions due to AMR range between 60.3 and 170.4 thousand per year, and the excess cost due to AMR in these admissions has been estimated to be approximately USD 0.5 million. However, the lower total cost of AMR in this context is indicative of resource constraints, leading to lower treatment intensity rather than a lower need for healthcare [10].

A recent report released by the Institute for Health Metrics and Evaluation (IHME) in 2025 shows that in 2021, the mortality rate due to AMR in South Africa, Lesotho, Eswatini, Botswana, Namibia, Angola, Zambia, Zimbabwe, Malawi, and Mozambique were 545, 1060, 875, 621, 749, 910, 1020, 1010, 915, and 1030 deaths per 100,000, respectively, with drug-resistant *K. pneumoniae* as the dominating Gram-negative pathogen (Table 1), accounting for approximately 4414 deaths [11].

Africa CDC purports that AMR may be a more significant health threat for Africa than HIV/AIDS, malaria, and tuberculosis and could reverse years of progress in healthcare [12]. In 2019 alone, sub-Saharan Africa recorded the highest rate of AMR burden, with 23.7 deaths per 100,000 people and 255,000 deaths attributed to AMR [12]. As opposed to high income countries where AMR is driven by overuse of antibiotics, in this context, AMR is driven by poor healthcare infrastructure, poor antimicrobial use policies, lack of antibiotic stewardship, and poor sanitation, leading to antimicrobial drug resistance rates that are 3–4 times higher compared to high-income settings [13].

Treatment options in infections caused by MDR bacteria are limited, with documented high levels of resistance to third-generation cephalosporins in Africa [14,15,16] and increasing levels of resistance to last-resort carbapenems globally [3]. Resistance to carbapenems in Gram-negative bacteria has increased globally more than any other antibiotic class, showing a 66% increase in the number of deaths associated with carbapenem resistance and a 70% increase in deaths attributable to carbapenem resistance from 1990 compared to 2021 [3]. This may be a due to high resistance levels to cephalosporins, which necessitated an increase in the use of carbapenems. Similarly, there was an increase in global mortality due to 3GC-resistant Enterobacterales in the above-mentioned time period [3]. These bacteria constitute the current critical group of the WHO priority pathogens list and highlight the urgent need for concerted efforts to minimize and control the development of multi-drug resistance.

β-lactam drugs are the most widely used antibiotics worldwide and include penicillins, cephalosporins, monobactams, and carbapenems [17]. In Gram-negative bacteria, β-lactamases remain the most important contributing factors to β-lactam resistance. This is exacerbated by their increasing frequency, especially in clinical isolates, and their continuous evolution [18]. Using the Ambler classification, which has widely been accepted in literature, the β-lactamases are classified A, B, C, and D according to their molecular structure [19]. Groups A, C, and D have a serine active center, while group B bears a zinc catalytic center [20]. Groups A and D have been found to be more prevalent in Africa [21]. These encompass the TEM β-lactamase (formerly referred to as Temoniera) (TEM); sulfhydryl variable β-lactamase (SHV); cefotaximase β-lactamase (CTX-M); class A carbapenemases, such as *Klebsiella pneumoniae* carbapenemase (KPC), Guiana extended-spectrum carbapenemase (GES), *Serratia marcescens* enzyme (SME), and imipenem hydrolyzing β-lactamase (IMI); oxacillinases (OXAs) and class D carbapenem-hydrolyzing β-lactamases [20,22]. The most prevalent ESBL gene globally is *CTX-M*, with the *CTX-M-15* variant being prevalent in Africa [21], especially in problematic Enterobacterales such as *E. coli* and *K. pneumoniae* [23].

Although some reports have associated ESBL/CPE determinants, such as *bla*_OXA–48_, with a virulent phenotype, a study showed that IncL (plasmids carrying *bla*_OXA–48_) transconjugants, generated from these highly virulent isolates, were not more cytotoxic or virulent when compared to the recipient strain [24]. Differences were only observed in the rate of horizontal gene transfer (HGT), where HGT was more frequent in isolates with the IncL plasmid but low in isolates with non-IncL plasmids [24]. However, a study investigating co-carriage of the CPE determinants *bla*_NDM-1_ and *bla*_OXA-232_ showed significantly increased bacterial fitness, virulence, and biofilm production [25]. The impact of these genetic variants on virulence and pathogenicity remain debatable and require further study. In contrast, the presence of the *bla_NDM-1_* gene only reduced the motility of *E. cloacae*, with no significant effect on their ability to resist serum killing or their virulence to cells [26]. In some instances, enhanced virulence and pathogenicity observed in isolates carrying ESBL/CPE determinants are due to the co-carriage of acquired virulence factors, in particular extended β-lactamase genes and carbapenemase genes located on the same hybrid plasmids [27].

Additionally, β-lactamases can be classified according to functional characteristics, which directly translate into clinical roles, including the differential and selective hydrolysis of β-lactam molecules [28]. Table 2 below shows the functional classification of β-lactamases, as well as the Ambler classification of different representative enzymes.

Despite the Africa CDC’s efforts to enhance antimicrobial resistance (AMR) surveillance through initiatives like the Africa Pathogen Genomics Initiative (Africa PGI) [12], significant gaps persist in the region’s capacity to effectively monitor and respond to AMR threats. The genetic determinants imparting multi-drug resistance are poorly characterized in this region, with lack of epidemiological data due to weak surveillance systems, poor diagnostic capacity and limitations in data collection and sharing [29]. Africa CDC reports that only 1.3% of laboratories in the continent are capable of performing bacteriological analysis for key pathogens, with the rest having low testing volumes, limited pathogen coverage, and lack of accreditation for bacteriology analyses [12]. In addition, the majority of available epidemiological data is highly variable, which impedes the management of AMR [29,30].

In a 2021 WHO systematic review, only 17 publications relating to AMR were published from South Africa, Lesotho, Eswatini, Botswana, Namibia Angola, Zambia, Zimbabwe, and Mozambique cumulatively [31], demonstrating the lack of AMR data in this region. Considering that Southern Africa is the region most heavily affected by the *HIV* epidemic, making an important part of its population more vulnerable to bacterial infections, we performed a literature review, consolidating the current knowledge on the epidemiology of ESBL- and carbapenemase-producing Enterobacterales in this region.

The aim of this narrative literature review was to summarize current knowledge on the epidemiology and molecular epidemiology of ESBL-producing Enterobacterales (ESBL-PE), carbapenemase-producing Enterobacterales (CPE), and carbapenem-resistant (CRE) in the Southern African countries, including South Africa, Lesotho, Eswatini, Botswana, Namibia, Angola, Zambia, Zimbabwe, Mozambique, and Malawi, while identifying critical gaps in the available literature.

## 2. Results

### 2.1. Epidemiology of ESBL-PE in Southern Africa

The reported prevalence of ESBL-PE varies widely depending on the context and source of isolates. Resistance to β-lactam antibiotics remains the most common, reflecting their widespread use in healthcare settings globally [32]. However, there is a notable lack of data on the prevalence and genetic determinants of resistance to β-lactam drugs in the Southern African region [33].

**Figure 1:**
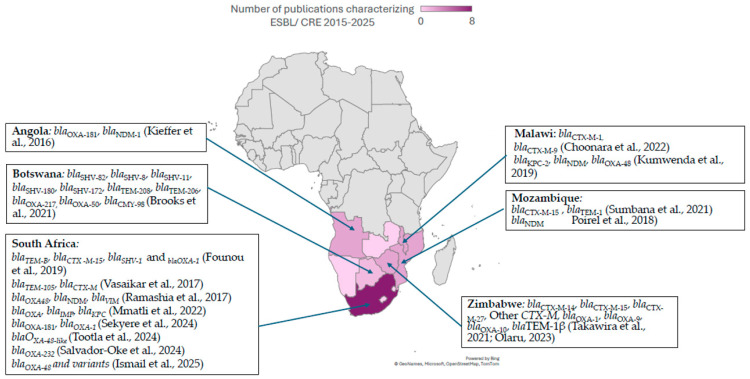
ESBL and CPE genetic determinants in Southern African countries, extracted from studies published between 2015 and 2025 [35,36,43,44,45,46,47,48,49,50,51,52,53,54,55,56].

In a recent meta-analysis, the prevalence of ESBL-producing *E. coli* in Southern Africa was 13.76%, which was the lowest in sub-Saharan Africa (SSA) [34]. However, this data included human, environmental, and animal isolates and shows that animals are the highest contributor to ESBL-type resistance in the SSA region. In addition, the most prevalent ESBL genetic determinant was *CTX-M-15* [34], which is consistent with several studies from Southern African countries, for example, a study from Zimbabwe characterizing *E. coli* isolated from urine specimens [35]. A prevalence study in South Africa reported the prevalence of ESBL-producing ESKAPE (*Enterobacter* spp., *S. aureus*, *K. pneumoniae*, *A. baumannii*, *P. aeruginosa*, *E. faecalis*), as 19.5%, with *K. pneumoniae* accounting for 29% of all isolates in the study [36]; see Figure 1 and Supplementary Table S1.

In a study performed in Namibia in 2017, the prevalence of ESBL-PE isolated from urine were 22%, 31.4%, and 8.3% for *E. coli*, *K. pneumoniae*, and *Proteus mirabilis*, respectively [37]. Maternal fecal carriage of ESBL-PE was 4.4% compared to 3.5% among infants in South Africa [38]. In Botswana, the prevalence of extended-spectrum cephalosporin-resistant (ESCr) isolates was similar between adults and children in the community, at 24% and 26%, respectively [39]. Among children living with HIV in Zimbabwe, ESBL-PE colonization was reported at 13.7% [40], further highlighting variability in carriage across populations. Colonization with ESCr Enterobacterales was higher in hospital settings in Botswana when compared to community settings. This was demonstrated in a study by Mannathoko et al. [39], where the prevalence of colonization by ESCrE was 30.7%. When stratified by setting, colonization with ESCrE was 43% for hospital participants, 31% for clinic participants, and 24% and 26% for adult and child community participants, respectively. A study performed in South Africa showed that colonization with ESBL-producing bacteria was associated with hospitalization, with colonization rates of 37.21%, 42.31%, and 57.14% at admission, after 48 h, and at discharge, respectively [41]. In Botswana, the risk factors for colonization and/or infection with ESBL-PE include exposure to a hospital setting, household transmission in instances where one or more member is colonized by ESCrE, tending to livestock on farms, and recent international travel [42]. See Figure 1 and Supplementary Table S1.

### 2.2. Epidemiology of CPE and CRE in Southern Africa

Carbapenemase-producing Enterobacterales (CPE) and carbapenem-resistant Enterobacterales (CRE) continue to be a significant healthcare threat, especially in low-middle income settings. The prevalence of CPE is highly variable between countries in Southern Africa. In a colonization study performed in Botswana, the prevalence of CPE was 1.7%, with the highest rates in hospital settings (6.8%) in comparison to community (0.2%) and clinic settings (0.7%). In the study, the most prevalent CPE was *E. coli* (n = 15), followed by *K. pneumoniae* (n = 14) [39].

These patterns are reflected in several studies, in which *K. pneumoniae* and *E. coli* dominate the CPE landscape, with isolation from rectal swabs, tracheal aspirates, urine, blood, and different sites of infections [43,44,45,46,47,48,49]. This was demonstrated in a study performed in South Africa by Ramashia et al. [49], which identified a carbapenem-resistant *K. pneumoniae* cluster in intensive care units (ICUs), surgical wards, and neonatal units. Additionally, there has been evidence of vertical and horizontal transfer of AMR genes, mainly mediated by IncX, IncF, and IncL plasmids [50]. Studies from South Africa demonstrated a concomitant carriage of carbapenem-resistance genetic determinants, for example, *bla*_OXA48_-producing isolates co-harboring *bla*_VIM_, *bla*_NDM_, *bla*_KPC_, and *bla*_IMP_ genes and carriage of *bla*_VIM_, *bla*_NDM_, and *bla*_OXA48_ by one *K. pneumoniae* isolate [48,49]. The prevalence of CPE in pediatrics, children, and adults varies substantially and may be influenced by sampling bias, from 36% from children in a pediatric hospital in Angola [51], to 0.2% and 0.5% in adult and children’s cohorts respectively, among Botswana community participants [39]. Risk factors for colonization, infection, and mortality due to CPE included being male, aged over 60 years, having pre-existing conditions, previously on antibiotics, mechanical ventilation, or catheterization, as well as oxygenation and previous hospital admission [52,57,58].

The genetic determinants of resistance to carbapenems are globally distributed, with variability between continents and regions [52]. However, there is a pronounced lack of direct country-specific molecular epidemiology data for several countries in Southern Africa, including Botswana, Lesotho, Eswatini, and Zimbabwe (Figure 1). In Southern African countries where molecular determinants of carbapenemase resistance have been characterized, group D *bla*_OXA-48_ and *bla-*_OXA48-like_ enzymes, as well as group B *bla*_NDM_, are most frequently detected. This pattern is consistent in studies from South Africa [59] and Angola [51,53], where there are at least two studies reporting the genomic characterization of CPE/CRE. In contrast, class B carbapenemase genes *bla_VIM_* and *bla_IMP_* and class A *bla*_KPC_ have been reported sporadically in South Africa, Mozambique, Angola, and Malawi (Figure 1 and Supplementary Table S2), often in the context of isolated outbreaks or single-center studies. This is a demonstration of global spread: KPC is traditionally endemic in the Americas, Middle East, Italy, Greece, and China, while *bla*_VIM_, which was first described in Italy, is endemic in Asia and Oceania and *bla*_IMP_, which was first identified in Japan, is still endemic in Southeastern Asia [60,61]. These studies suggest that the epidemiological landscape of CPE/CRE genetic determinants mirrors global distributions, with *bla_OXA_* and *bla_NDM_* dominating the landscape in Southern Africa.

### 2.3. Available Molecules for Treatment of Infections Due to MDR

Treatment options for infections caused by multidrug-resistant *Enterobacterales* remain severely limited, with currently available agents providing limited empirical coverage [54,55,56,62]. This highlights the importance of establishing local antimicrobial susceptibility patterns and the epidemiology of ESBL- and carbapenemase-producing and Enterobacterales to guide empirical therapy.

In response to rising resistance to cephalosporins and carbapenems, several novel agents have been developed. These include ceftazidime/avibactam, meropenem/vaborbactam, ceftolozane/tazobactam, plazomicin, and eravacycline [63]. However significant challenges remain. One of the key limitations of novel β-lactam/β-lactamase inhibitor combinations is their variable activity depending on the specific carbapenemase involved. For instance, ceftazidime/avibactam exhibits activity against class A and some class D carbapenemases, whereas imipenem/relebactam is active against class A and class C enzymes but lacks efficacy against metallo-β-lactamases (MBLs) and class D carbapenemases [64,65]. However, emerging resistance to these new agents has been reported [66]. The principal mechanisms related to the resistance to novel antimicrobial molecules are summarized in Table 3, including βL/βLI combinations, β-lactam/non-β lactamase inhibitor (βL/nβLI), and aminoglycosides, their spectrum of activity, as well as molecular determinants of resistance.

In detail, resistance to ceftazidime/avibactam has often been associated with mutations (i.e., (insertions, deletions, and/or point mutation) within carbapenemase genes, and in particular the *bla*_KPC_ gene [66,67]. In particular, three hotspot regions have been described within the *bla*_KPC_ gene, resulting in ceftazidime/avibactam resistance. Other mechanisms observed less frequently among Enterobacterales and associated with ceftazidime/avibactam resistance include: altered outer membrane porins, increased gene expression and copy number of carbapenemase genes, mutations within B-lactamase genes (*bla*_CTX-M_, *bla*_SHV_, and Amp C), and overexpression of efflux systems.

Resistance to meropenem/vaborbactam is mainly associated with impaired permeability due to porin mutations associated with overexpression of β-lactamase and increases in efflux pump production [66,68].

For imipenem/relebactam, resistance has been associated with carbapenemase mutation, carbapenemase over-expression, penicillin-binding protein (PBP) mutation or under-expression, increased efflux, and decreased permeability [66].

Resistance to cefiderocol is associated with different mechanisms, often combined, which contribute to reduced cefiderocol susceptibility [66]. Among them, the most common mechanisms include β-lactamases, mutations affecting expression/function of siderophore receptors (most commonly involved *cirA* and *fiu* genes in Enterobacterales), mutations resulting in the expression/function of porins and/or efflux pumps, or target modification.

Resistance to cefepime/taniborbactam is mainly due to multiple β-lactamase production, target alterations, porin loss, and efflux pump upregulation).

For aztreonam/avibactam, resistance has been associated with target mutations, co-production of ESβL, and dual-carbapenemase expression.

Lastly, emerging resistance to novel antimicrobial molecules against ESBL-PE, CRE, and CPE include resistance to eravacycline due to plasmid-borne gene or efflux pumps/mutations and plazomicin due to target site modification and enzymatic inactivation.

Data on the registration and clinical use of novel BL/BLI in Southern African countries is scant, with only a report of registration and use of ceftolozane/tazobactam and ceftazidime/avibactam registered for use in South Africa in 2022 [87]. There are concerns of unregulated use, especially in the private sector and general use for any difficult-to-treat infections, which may drive possible resistance.

## 3. Discussion

ESBL-PE, CRE, and CPE represent a growing healthcare challenge in Southern African countries. The prevalence, as shown in this review, is highly variable according to the context: clinical vs. environmental isolates, human vs. animal isolates, and pediatric vs. adult population isolates. This is possibly influenced by several factors, including sampling strategies, community and healthcare exposure, and antibiotic pressure. *Klebsiella pneumoniae* and *E. coli* dominate the ESBL-PE, CRE, and CPE landscape, frequently being isolated from urine, stool, and blood at clinical sites. While the detection of some genetic determinants of ESBL or carbapenem resistance show low prevalence, such as *bla_KPC_*, the landscape mirrors global epidemiology, with the most prevalent β-lactamase determinants being *bla*_CTX-M_, and *bla*_NDM_ and *bla*_OXA_ being the most prevalent carbapenemase enzymes [88,89,90,91,92,93].

Despite growing recognition of the relevance of these resistance mechanisms, molecular characterization is still scant, with countries such as Botswana, Lesotho, Eswatini, Zambia, and Zimbabwe lacking published information describing the prevalence and genetic diversity of ESBL-PE, CRE, and CPE. This lack of data impedes the development of local empiric therapy, as the effectiveness of antibiotic interventions depends on the functional classification of the resistance determinants [65]. The development of novel antibiotics represents an important advancement in combating ESBL-PE, CRE, and CPE. In the context of this narrative literature review, there is limited information on the use of these agents in most countries, with regulatory and utilization information available only for South Africa. In addition, the usage of novel antibiotics in private facilities may raise some concern about the emergence of resistance to these new therapeutic options. In summary, addressing these healthcare challenges hinges on coordinated efforts in molecular epidemiology, antibiotic stewardship, infection prevention and control, and more importantly, equitable access to therapeutic options.

The current study has some limitations. First, data availability for colonization, therapeutical outcomes, and the genetic determinants of resistance are not available/consistent for most countries, with the exception of South Africa. Second, publication bias and data heterogeneity should be taken into consideration, despite our efforts to cover the subject as comprehensively as possible.

## 4. Materials and Methods

This narrative literature review consulted PubMed and Web of Science for primary information. Furthermore, Google Scholar was also consulted for completeness. We used the search prompt (“ESBL” OR “Extended-spectrum β-lactamase” OR “CPE” OR “Carbapenemase-producing Enterobacterales” OR “CRE” OR “Antibiotic resistance”) AND (“epidemiology” OR “molecular epidemiology” OR “genotypic characterization” OR “genetic diversity” OR “molecular typing” OR “whole genome sequencing”) AND (“South Africa” OR “Lesotho” OR “Eswatini” OR “Swaziland” OR “Botswana” OR “Namibia” OR “Angola” OR “Zambia” OR “Zimbabwe” OR “Mozambique” OR “Malawi”). It should be clarified here that the Kingdom of Swaziland officially changed its name to the Kingdom of eSwatini in April 2018, and we took this into consideration in our search criteria All publications that matched the key words were included. The titles of the references were scanned for keywords matching our selection criteria and were included if they met at least one of them. We only included studies performed using isolates in the Southern African countries of South Africa, Lesotho, Eswatini, Botswana, Namibia, Angola, Zambia, Zimbabwe, Mozambique, and Malawi. Two authors (PN and GMP) independently reviewed the titles, abstracts, and full articles of the retrieved papers. We included studies that characterized isolates from human samples; when data included human, animal, and environmental isolates with no separate analysis, this was acknowledged. Grey literature, such as postgraduate dissertations, were also included. The search period was from June 2025 to September 2025 and included studies published in the English language from the year 2015 to the year 2025.

## 5. Conclusions

While the available evidence highlights important trends in species distribution and resistance determinants, significant knowledge gaps persist, especially in countries with limited diagnostic capacity. These gaps challenge the development of effective, context-specific treatment guidelines. Therefore, strengthening surveillance systems, expanding molecular characterization, and ensuring equitable access to both existing and novel antimicrobial agents are essential. Additionally, coordinated regional efforts that integrate laboratory capacity strengthening and antimicrobial stewardship are critical to mitigating the clinical and public health impact of ESBL-PE, CRE, and CPE in Southern Africa, as well as other regions in the continent.

## Figures and Tables

**Table 1:** Country-specific leading Enterobacterales pathogen–drug combinations and estimated mortality attributed to antimicrobial resistance according to data from 2021 [8].

Country	Leading Pathogen–Drug Combination (Enterobacterales) and Attributed Mortality
Angola	Aminoglycosides-resistant *K. pneumoniae* (206, UI 131–281) Fluoroquinolones-resistant *K. pneumoniae* (151, UI 86–217)
Botswana	TMP/SMX-resistant *K. pneumoniae* (11, UI 5–17) 3GC-resistant *E. coli* (9, UI 3–15) TMP/SMX-resistant *E. coli* (8, UI 5–12)
Eswatini	3GC-resistant *K. pneumoniae* (10, UI 5–14) Aminoglycosides-resistant *K. pneumoniae* (7, UI 4–10) Fluoroquinolone-resistant *K. pneumoniae* (7, UI 4–10) Fluoroquinolone-resistant *E. coli* (5, UI 2–8)
Namibia	TMP/SMX-resistant *K. pneumoniae* (16, UI 8–25) βL/βLI-resistant *K. pneumoniae* (10, UI 2–19) βL/βLI-resistant *E. coli* (10, UI 3–17) TMP/SMX-resistant *E. coli* (9, UI 5–12)
Lesotho	Fluoroquinolone-resistant *K. pneumoniae* (21, UI 12–30) TMP/SMX-resistant *K. pneumoniae* (17, UI 9–26)
South Africa	Carbapenem-resistant *K. pneumoniae* (447, UI 351–544) Fluoroquinolone-resistant *K. pneumoniae* (301, UI 208–394) Aminoglycoside-resistant *K. pneumoniae* (285, UI 201–370) TMP/SMX-resistant *K. pneumoniae* (253, UI 129–376)
Malawi	3GC-resistant-*K. pneumoniae* (200, UI 113–286) Carbapenem-resistant *K. pneumoniae* (145, UI 101–190) Aminoglycoside-resistant *K. pneumoniae* (133, UI 90–176) Fluoroquinolone-resistant *K. pneumoniae* (114, UI 70–158) TMP/SMX-resistant *K. pneumoniae* (110, UI 55–165) 3GC-resistant *E. coli* (99, UI 35–162)
Mozambique	3GC-resistant *K. pneumoniae* (358, UI 197–519) Fluoroquinolone-resistant *K. pneumoniae* (260, UI 157–362) TMP/SMX-resistant *K. pneumoniae* (219, UI 106–332) TMP/SMX-resistant *E. coli* (185, UI 124–247) Aminoglycoside-resistant *K. pneumoniae* (148, UI 92–203)
Zambia	3GC-resistant *K. pneumoniae* (219, UI 115–324) Fluoroquinolone-resistant *K. pneumoniae* (132, UI 79–184) 3GC-resistant *E. coli* (120, UI 53–187) TMP/SMX-resistant *K. pneumoniae* (114, 55–173) TMP/SMX-resistant *E. coli* (102, 67–137) Fluoroquinolone-resistant *E. coli* (98, 49–146)
Zimbabwe	3GC-resistant *K. pneumoniae* (182, UI 106–258) TMP/SMX-resistant *K. pneumoniae* (158, UI 80–237) Carbapenem-resistant *K. pneumoniae* (96, UI 69–123) Aminoglycoside-resistant *K. pneumoniae* (84, 54–114)

**Table 2:** Overview of β-lactamases according to molecular classes (Ambler classification), functionality, and representative enzymes.

Ambler Classification	Functional Classification (Substrates in Parentheses)	Enzymes
Class A	*Group 2 serine β-lactamases* 2a (penicillin) 2b (penicillin) 2be (extended-spectrum cephalosporins, monobactams) 2br (penicillin) 2ber (extended-spectrum cephalosporins, monobactam) 2c (carbenicillin) 2ce (carbenicillin, cefepime) 2e (extended-spectrum cephalosporins) 2f (carbapenems)	TEM-1 TEM-2 SHV-1 TEM-3 SHV-2 CTX-M-15 TEM-30 SHV-10 TEM-50 KPC-2 IMI-1 SME-1
Class B	*Group 3 metallo-βlactamases and substrates* 3a (carbapenems) 3b (carbapenems)	IMP-1 VIM-1 IND-1 SFH-1
Class C	*Group 1 cephalosporinases and substrates* 1 (cephalosporins) 1e (cephalosporins)	AmpC CMY-2 CMY-37
Class D	*Group 2 serine β-lactamases and substrates* 2d (cloxacillin) 2de (extended-spectrum cephalosporins) 2df (carbapenems)	OXA-1 OXA-10 OXA-11 OXA-15 OXA-23 OXA-48

**Table 3:** Novel antibiotics, spectrum of activity, and molecular determinants of resistance.

Novel Antimicrobial Agent	Spectrum of Activity	Resistance
Ceftazidime/avibactam	Class A, class C, class D carbapenemase-producing Enterobacterales [67].	Cases of resistance conferred by *bla*_KPC_ mutations have been reported in Enterobacterales [67].
Meropenem/vaborbactam	Activity against class A and class C β-lactamases [68].	Resistance conferred by increased *bla*_KPC_ copy number and mutations in the ompK36 porin in *K. pneumoniae* [69].
Ceftolozane/tazobactam	Active against ESBL producers and class A lactamases [70].	Resistance observed in KPC- and MBL-producing Enterobacterales [71].
Plazomicin	Broad spectrum activity against β-lactamase- and carbapenemase-producing Enterobacterales [72].	Possible resistance described in NDM-1-producing CRE strains, conferred by co-expression of 16S ribosomal plazomicin-inactivating methyltransferases in Enterobacterales [73].
Eravacycline	Broad spectrum of activity including in CRE and bacteria resistant to β-lactam/β-lactamase inhibitors [74]. Activity against class A, class B, and class D β-lactamases [75]	Resistance mediated by mutations in the Lon protease, upregulation of AcrAB-TolC, and porins OmpA and OmpU in Enterobacterales [76].
Cefepime/taniborbactam	Inhibits classes A, C, and D and MBLs, including NDM and VIM, but not IMP [77].	Resistance in Enterobacterales arises due to the concurrent presence of multiple resistance mechanisms. For example, production of IMP, alterations in PBP3, permeability defects, and upregulation of efflux pumps [78].
Imipenem/funobactam	Active against serine β-lactamases (class A, class C, and class D) [79].	No resistance mechanisms reported to date.
Cefiderocol	Highly active against all classes of carbapenemase. Class A, class B, class C, and class D β-lactamases [80].	Resistance in carbapenem-resistant Enterobacterales is mediated by different mechanisms. For example, in carbapenem-resistant *K. pneumoniae*, is mediated by CirA deficiency and the presence of metallo- or serine β-lactamases while in *E. coli*, it is due to mutations in PBP3 [81].
Imipenem/relebactam	Primarily restores clinical activity of imipenem against imipenem-resistant isolates Enterobacteriaceae. Inhibits Class A and B β-lactamases [82]	Does not restore susceptibility in Enterobacterales-producing OXA-48 carbapenemases, VIM, IMP, and NDM β-lactamases [83].
Aztreonam/avibactam	Active against Enterobacterales that produce MBLs [84]	Resistance may be conferred by unique mutations in β-lactamases, for example, Arg244gly substitution in *blaSHV12* [85].
Cefepime/zidebactam	Active against β-lactamase producing Enterobacterales including isolates that produce MBLs and Class D β-lactamases [86]	Possible resistance in ST14, OXA-232 producing *K. pneumoniae* [87].

## Data Availability

Not applicable.

## Supplementary Materials

The following supporting information can be downloaded at https://www.mdpi.com/article/10.3390/antibiotics15010069/s1, Table S1: Summary of Enterobacterales isolates analyzed by Southern African country, showing presence and diversity of extended-spectrum-β-lactamase genetic determinants; Table S2: Summary of Enterobacterales isolates analyzed by Southern African country, showing presence and diversity of CPE genetic determinants.

## Abbreviations

The following abbreviations are used in this manuscript:

3GC	Third-generation cephalosporins
AMR	Antimicrobial resistance
βL/βLI	Β-lactam/β lactamase inhibitor
βL/nβLI	Β-lactam/non-β lactamase inhibitor
CRE	Carbapenem-resistant Enterobacterales
CPE	Carbapenemase-producing Enterobacterales
CTX	Cefotaximase β-lactamase
ESBL	Extended-spectrum β-lactamase
ESCrE	Extended-spectrum cephalosporin-resistant Enterobacterales
GAP	Global action plan
GES	Guiana extended-spectrum carbapenemase
IMI	Imipenem hydrolyzing β-lactamase
KPC	*Klebsiella pneumoniae* carbapenemase
MBL	Metallo-β lactamase
MDR	Multi-drug-resistant
NAP	National action plan
OXA	Oxacillinases
SHV	Sulfhydryl variable β-lactamase
SME	*Serratia marcescens* enzyme (SME)
TEM	Temoneira
TMP/SMX	Trimethoprim-sulfamethoxazole
