# Supplementary material for: Epidemiology of ESBL-Producing, Carbapenem-Resistant, and Carbapenemase-Producing Enterobacterales in Southern Africa
Source: Antibiotics (Basel). 2026 Jan 8;15(1):69. doi: 10.3390/antibiotics15010069 (PMC12838327; doi:10.3390/antibiotics15010069)
Supplement: Supplementary file 1 [file antibiotics-15-00069-s001.zip › antibiotics-3958258-supplementary.pdf]

Country	Isolates	ESBL genetic determinants	Sequence types	Study design
South Africa	*K. pneumoniae* (n=9)	$bla_{\text{TEM-B}}$ (100%), $bla_{\text{CTX -M-15}}$ (100%), $bla_{\text{SHV-1}}$ (89%) and *blaOXA-1* (44.5%) [36].	ST607: $bla_{\text{TEM-1B}}$, $bla_{\text{CTX -M-15}}$, $bla_{\text{SHV-1}}$<br>ST17: $bla_{\text{TEM-1B}}$, $bla_{\text{CTX -M-15}}$, $bla_{\text{SHV-11}}$<br>ST32: $bla_{\text{TEM-1B}}$, $bla_{\text{CTX -M-15}}$, $bla_{\text{SHV-60}}$<br>ST983 *(n=2)*: $bla_{\text{TEM-1B}}$, $bla_{\text{CTX -M-15}}$, $bla_{\text{SHV-38}}$, $bla_{\text{SHV-168}}$<br>ST152 *(n=4)*: $bla_{\text{TEM-1B}}$, $bla_{\text{CTX -M-15}}$, $bla_{\text{SHV-1}}$, $bla_{\text{oxa-1}}$	*Observational prevalence study*
	K. pneumoniae (n=169), *K. oxytoca* (n=29), *K. ozaenae* (n=2)	bla_{SHV} (77.1%), $bla_{\text{TEM-105}}$ (66.9%), $bla_{\text{CTX-M}}$ (56.7%) [43].	No sequence type data.	*Prospective descriptive study*
Botswana	No isolate-specific information provided in the cited study	$bla_{\text{SHV-82}}$, $bla_{\text{SHV-8}}$, $bla_{\text{SHV-11}}$, $bla_{\text{SHV-180}}$, $bla_{\text{SHV-172}}$, $bla_{\text{TEM-208}}$, $bla_{\text{TEM-206}}$, $bla_{\text{OXA-217}}$ $bla_{\text{OXA-50}}$, $blaS_{\text{ED1}}$, $bla_{\text{OKP-B-17}}$ $bla_{\text{CMY-98}}$, $bla_{\text{LEN-24}}$ [44].	No sequence type data.	*Cross-sectional, observational genomic surveillance study*
Lesotho	-	No published data describing the epidemiology of ESBL genetic determinants.	-	-
Eswatini	-	No published data describing the epidemiology of ESBL genetic determinants.	-	-
Namibia	-	No published data describing the epidemiology of ESBL genetic determinants.	-	-
Angola	-	No data for clinical isolates. Data only for non-clinical niches.	-	-
Zambia	-	No published data describing the epidemiology of ESBL genetic determinants.	-	-
Zimbabwe	*E. coli* (n= 48)	$bla_{\text{CTX-M-14}}$ (6%), $bla_{\text{CTX-M-15}}$ (70%), $bla_{\text{CTX-M-27}}$ (23%), Other *CTX-M* (6%), $bla_{\text{OXA-1}}$ (54%), $bla_{\text{OXA-9}}$ (2%), $bla_{\text{OXA-10}}$ (2%), *bla*TEM-1β (58%) [35].	ST131 (n=19): $bla_{\text{CTX -M-15}}$, $bla_{\text{CTX -M-27}}$, Other CTX-M, bla_{OXA}, $bla_{\text{TEM-1B}}$<br>ST23 (n=14): $bla_{\text{CTX -M-14}}$, $bla_{\text{CTX -M-15}}$, bla_{OXA}, $bla_{\text{TEM-1B}}$<br>ST10 (n=3): $bla_{\text{CTX -M-15}}$, bla_{OXA}, $bla_{\text{TEM-1B}}$<br>ST405 (n=2): $bla_{\text{CTX -M-14}}$, $bla_{\text{CTX -M-15}}$, bla_{OXA}, $bla_{\text{TEM-1B}}$	*Observational genomic surveillance study*

	E. coli (n=223)	$bla_{CTX-M-15}$ (14%), $bla_{CTX-M-14}$ (1%), $bla_{CTX-M-27}$ (4%), $bla_{CTX-M-55}$ (<1%) [45].	ST648 (n=2): $bla_{CTX-M-15}$, bla_{OXA}, bla_{TEM-1B}<br>ST69: bla_{TEM-1B}<br>ST354: $bla_{CTX-M-15}$, $bla_{CTX-M-27}$, bla_{TEM-1B}<br>ST155: bla_{OXA}, bla_{TEM-1B}<br>UNKNOWN ST (n=5): $bla_{CTX-M-14}$, $bla_{CTX-M-15}$, Other CTX-M, bla_{TEM-1B}<br>Incomplete/ partial ST data.	*Observational genomic surveillance study*
Malawi	*E. coli* (n=38)	$bla_{CTX-M-15}$ *(85%)* [46].	ST104 (n=16): $bla_{CTX-M-15}$, bla_{TEM-1}, bla_{OXA-1}<br>ST131 (n=7): $bla_{CTX-M-15}$, $bla_{CTX-M-27}$, bla_{TEM-1}<br>ST617 (n=6): $bla_{CTX-M-15}$, bla_{TEM-1}, bla_{OXA-1}<br>ST155: $bla_{CTX-M-15}$, bla_{TEM-1}<br>ST48: $bla_{CTX-M-15}$, blaTEM-1<br>ST6332: $bla_{CTX-M-15}$, bla_{TEM-1}<br>ST354: $bla_{CTX-M-27}$, bla_{TEM-1}<br>ST5824: $bla_{CTX-M-14}$<br>ST38: $bla_{CTX-M-15}$, bla_{TEM-1}<br>ST44: $bla_{CTX-M-15}$, bla_{TEM-1}, bla_{OXA-1}<br>ST648: $bla_{CTX-M-15}$, bla_{TEM-1}, bla_{OXA-1}	*Observational genomic surveillance study*
	K. pneumoniae (n=17)		ST101 (n= 2): $bla_{CTX-M-15}$, bla_{SHV}, bla_{OXA}<br>ST1047): $bla_{CTX-M-15}$, bla_{SHV}, bla_{TEM-1}, bla_{OXA}<br>ST14 (n= 2): $bla_{CTX-M-15}$, bla_{SHV}, bla_{TEM-1}, bla_{OXA}<br>ST15: $bla_{CTX-M-15}$, bla_{SHV}, bla_{TEM-1}, bla_{OXA}<br>ST1552: $bla_{CTX-M-15}$, bla_{SHV}, bla_{TEM-1}<br>ST231: $bla_{CTX-M-15}$, bla_{SHV}, bla_{TEM-1}, bla_{OXA}	

			ST29: *bla*CTX-M-15, *bla*SHV, *bla*TEM-1, *bla*OXA<br>ST307: *bla*CTX-M-15, *bla*SHV, *bla*TEM-1<br>ST340 (n= 2): *bla*CTX-M-15, *bla*SHV, *bla*TEM-1, *bla*OXA<br>ST48: *bla*CTX-M-15, *bla*SHV, *bla*TEM-1, *bla*OXA<br>ST607: *bla*CTX-M-15, *bla*SHV<br>ST874: *bla*CTX-M-15, *bla*SHV, *bla*TEM-1, *bla*OXA<br>UNKNOWN ST (n= 2): *bla*CTX-M-15, *bla*SHV, *bla*TEM-1, *bla*OXA	
Mozambique	*E. coli* (n=1)	*bla*CTX-M-15 (100%)<br>*bla*TEM-1 (100%) [47].	ST405: *bla*CTX-M-15, *bla*TEM-1	*Case report*

Supplementary Table 1. Summary of Enterobacterales isolates analyzed by Southern African country, showing presence and diversity of extended-spectrum-β-lactamase genetic determinants.

Country	Isolates	Genetic determinants of CPE	Sequence types	Study design
South Africa	*K. pneumoniae* (n= 82)	*bla*OXA48 (65%), *bla*NDM (25%), *bla*VIM (22%) [49].	*No sequence type data*	*Retrospective, observational genomic surveillance study*
	K. pneumoniae (n=208)<br>*E. coli* (n=29)<br>*E. cloacae complex* (n=16)<br>*K. oxytoca* (n=3)<br>*S. marcescens* (n=2)<br>*C. freundii* (n=1)<br>*C. koseri* (n=1)<br>*Other* (n= 45)	*bla*OXA (84%), *bla*NDM (15%), *bla*IMP (5.2%), *bla*VIM (5.2%), *bla*KPC (4.1%) [48].	*No sequence type data*	*Retrospective, observational surveillance study.*
	K. pneumoniae (79%)<br>*E. coli* (8%),	*bla*OXA48 (44%), *bla*NDM (38%), *bla*OXA-181 (29%), *bla*OXA-1 (12%) [50]	ST5785: *bla*OXA, *bla*VIM<br>ST25: *bla*OXA<br>ST307 (n=8): *bla*OXA (88%)<br>ST152 (n=2): *bla*OXA, *bla*NDM (100%)<br>ST152 (n=10): *bla*NDM (100%)<br>ST17 (n=2): *bla*OXA (100%)	*Retrospective outbreak investigation*

	E. cloacae (8%)<br><br>*Citrobacter* (3%).		ST907: *bla*$_{\mathrm{OXA}}$<br>ST906: *bla*$_{\mathrm{OXA}}$<br>ST58: *bla*$_{\mathrm{OXA}}$<br>ST457: *bla*$_{\mathrm{OXA}}$<br>ST829: *bla*$_{\mathrm{OXA}}$<br><br>ST1266 (n=3): *bla*$_{\mathrm{OXA}}$<br><br>ST248: *bla*$_{\mathrm{OXA}}$	
	Klebsiella species (n=87)<br>*K. pneumoniae* (n=85)<br>*K. oxytoca* (n=1)<br>*K. variicola* (n=1)<br>*S. marcescens* (n=13)<br>*E. cloacae* (n=8)<br>*C. freundii* (n=5)<br>*E. coli* (n=2)<br>*P. rettgeri* (n=2)	*bla*$_{\mathit{OXA\text{-}48\text{-}like}}$ (80%), *bla*$_{\mathrm{NDM}}$ (11%) [59]	*No sequence type information.*	*Prospective, observational surveillance study*
	K. pneumoniae (n= 446)	*bla*$_{\mathrm{NDM}}$ (33%), *bla*$_{\mathrm{OXA\text{-}181}}$ (30%), *bla*$_{\mathrm{OXA\text{-}232}}$ (15%) [60]	*Sequence type information for 22 isolates.*<br>ST17 (n=4): *bla*$_{\mathrm{OXA\text{-}181}}$<br>ST30: *bla*$_{\mathrm{OXA\text{-}181},}$ *bla*$_{\mathrm{KPC\text{-}2}}$<br>ST34: *bla*$_{\mathrm{VIM}}$<br>ST307: *bla*$_{\mathrm{OXA\text{-}181},}$ *bla*$_{\mathrm{NDM},}$ *bla*$_{\mathrm{OXA\text{-}48}}$<br>ST147: *bla*$_{\mathrm{OXA\text{-}181}}$<br>ST2497 (n=5): *bla*$_{\mathrm{NDM},}$ *bla*$_{\mathrm{OXA\text{-}232}}$<br>ST147: *bla*$_{\mathrm{NDM}}$<br>ST152 (n=2): *bla*$_{\mathrm{OXA\text{-}48},}$ *bla*$_{\mathrm{NDM\text{-}1}}$<br>ST2795: *bla*$_{\mathrm{OXA\text{-}181}}$	*Descriptive molecular characterization study*

	K. pneumoniae<br>*E. cloacea*<br>*S. marcescens*<br>*E. coli*<br>*Other* spp.	*bla*OXA-48 and variants (71%), b*la*NDM (28%), *bla*VIM (2%) [54]	ST147: *bla*NDM-1<br>ST353: *bla*KPC-2<br>ST152: *bla*NDM, *bla*OXA-48<br>ST307 (n=2): *bla*OXA-181<br><br>No sequence type information.	*Cross-sectional study*
Botswana	-	No published data describing the epidemiology of CPE genetic determinants.	-	-
Namibia	-	No published data describing the epidemiology of CPE genetic determinants.	-	-
Zimbabwe	-	No published data describing the epidemiology of CPE genetic determinants.	-	-
Zambia	-	No published data describing the epidemiology of CPE genetic determinants.	-	-
Angola	*E. coli* (n= 29)<br>*K. pneumoniae* (n=25)<br>*E. cloacea* (n=1)<br>*Providencia spp.* (2)<br><br>*E. coli* (n=9)<br>*K. pneumoniae* (n=8)<br>*Enterobacter spp.* (n=4)<br>*Citrobacter spp.* (n=1)	*bla*OXA-181 (87.7%), *bla*NDM-1 (12%) [51]<br><br>*bla*NDM-5 (45%) [56]		*Cross-sectional study*<br><br>*Prospective colonization study*
Mozambique	*E. coli* (n=1)	*bla*NDM [47]	ST405*: bla*NDM (100%)	*Case report*
Malawi	*Klebsiella spp.*<br>*E. coli*	*bla*KPC-2, *bla*NDM, *bla*OXA-48 [61]		*Retrospective, observational surveillance*

Lesotho	-	No published data describing the epidemiology of CPE genetic determinants.	-	-
Eswatini	-	No published data describing the epidemiology of CPE genetic determinants.	-	-

Supplementary Table 2. Summary of Enterobacterales isolates analyzed by Southern African country, showing presence and diversity of CPE genetic determinants.
